# Supplementary material for: Are carbon emissions trading and green financial instruments synergistic? -Comprehensive quantitative research based on content analysis
Source: PLoS One. 2024 Mar 7;19(3):e0298601. doi: 10.1371/journal.pone.0298601 (PMC10919601; doi:10.1371/journal.pone.0298601)
Supplement: S1 Data — (ZIP) [file pone.0298601.s001.zip › Data/地级市Z_F工作报告.zip!/║ú─╧/╚2╤╟╩╨/└┤╫╘╣1⁄2╓┌║┼í╛┬φ┐╦╩2╛▌═°í┐_2011.docx]

各位代表：

现在，我代表市人民政府向大会作工作报告，请各位代表连同《三亚市国民经济和社会发展第十二个五年规划纲要(草案)》一并审议，并请市政协各位委员和其他列席人员提出意见。

2010年主要工作回顾

2010年是国际旅游岛建设开局之年，也是“十一五”规划收官之年。一年来，市政府坚持以科学发展观为指导，在市委的正确领导下，在市人大、市政协的监督支持下，紧紧围绕国际旅游岛建设，科学开展规划部署，扎实推进各项工作，克服了强降雨灾害等不利因素的影响，努力打造中外游客的度假天堂和三亚人民的幸福家园，取得了较好的发展成绩。全市经济保持平稳快速发展，社会事业全面进步，人民生活不断改善，顺利完成了市五届人大五次会议确定的年度任务。

――经济实力进一步增强。初步核算，2010年全市实现生产总值230.8亿元（不含农垦，下同），同比增长20.2%（按可比价格计算，下同）。全社会固定资产投资305.2亿元，增长44.4%；其中，全市107个重点项目共完成投资187.3亿元。全市社会消费品零售总额63.7亿元，增长25%。全年累计实现进出口总额5297万美元，增长12%；其中，出口5147万美元，增长10.7%。

――旅游产业龙头作用进一步凸显。全市游客接待量特别是入境游客接待量实现了较大幅度的增长。2010年全市接待过夜游客人数882.7万人次，其中旅游饭店接待过夜游客751.1万人次，同比增长12.3%；全市接待过夜入境游客41.5万人次，增长30.6％。全市实现旅游总收入139.6亿元，按旅游饭店统计口径同比增长22.3%；其中，旅游外汇收入2.45亿美元，增长25.5%。

――发展方式转变进一步加快。产业结构优化取得新成效，2010年我市第一、二、三产业分别完成增加值31亿元、49.4亿元、150.4亿元，分别拉动GDP增长1个、5个、14.2个百分点。节能减排、生态环保工作取得新进步，顺利完成了省下达的单位生产总值能耗指标以及化学需氧量、二氧化硫排放量指标，生态环境质量继续保持全国一流，可持续发展能力持续增强。

――发展质量进一步提升。按户籍人口计算，2010年全市人均生产总值46624元，同比增长17.7%。全市地方财政一般预算收入42.2亿元，增长58%；其中，税收收入38.3亿元，增长62.6%。截至去年12月底，全市金融机构本外币各项存款余额616.4亿元，贷款余额228.7亿元，分别比去年初增长28.9%、56.7%，不良贷款率仅为0.75%。金融机构整体盈利比上年同期增长64.7%。

――人民生活进一步改善。加快发展民生事业，全年拨付重点民生项目资金29.5亿元，同比增长39.2%。其中，住房保障、教育、医疗卫生、农林水事务支出分别增长78.6%、42.5%、35.2%、35.3%。全市城镇居民人均可支配收入17758元，增长16.6%；农民人均纯收入6502元，增长15.7%。全市城镇从业人员基本养老保险参保率达到98.99％，新型农村社会养老保险参保率达到95.6%，城乡居民基本医疗保险参保率达到99.98％。

一年来，我们主要做了以下几个方面的工作：

一、积极推进国际旅游城建设，打造旅游度假天堂。海棠湾、鹿回头半岛等片区开发进展顺利，其中海棠湾全年完成投资68.2亿元，规划区域内已有12条市政主干道通车，万达康莱德、希尔顿逸林和中港万丽3家酒店已建成试营业，解放军总医院海南分院主体结构工程已全部封顶。积极开展三亚河游艇基地建设和邮轮母港前期工作，基本完成了三亚湾旅游商业街改建工程，市内免税店三期营业区域已全部对外营业，境外旅客购物离境退税政策试点已在夏日百货有序实施。大东海等7个游客到访服务站已建成启用，“天涯通”系列服务产品陆续投入使用。从去年初开始，大力开展旅游环境综合整治，并在此基础上全力备战今年春节“黄金周”，认真分解工作任务和责任，建立立体式网格化管理体系。春节期间，各区镇、各有关部门24小时值班，公安交警全员上路，重点单位全员上班，全市交通拥堵状况明显改善，环境卫生大为优化，酒店房价回归理性，实现了海鲜排档欺客宰客零投诉和一日游零投诉，在游客接待量超过去年春节“黄金周”的情况下，保持了旅游接待工作平稳有序，得到了国家旅游局、省委、省政府和社会各界的充分肯定。

二、积极推进产业结构优化调整，加快转变经济发展方式。在国家宏观调控政策的指导下，努力保持房地产业健康发展，2010年全市房地产开发投资132.8亿元，商品房销售面积140.8万平方米。创意产业园等园区基础设施建设稳步推进，项目招商顺利开展，目前创意产业园已签约24个项目，海南动漫产业基地已引入16家企业。大力推进“万村千乡市场”工程，建成了三亚国际购物中心、天成超市新风路店并投入营业，消费品市场保持活跃。积极培育文化体育产业，奥林匹克湾、沃尔沃帆船赛经停港基地、崖城历史文化名镇保护与整治等项目稳步推进，成功举办了第60届世界小姐总决赛等文化体育赛事。大力发展冬季瓜菜、热带水果、花卉、南繁育种等热带特色产业，建设了近20个农业示范基地，稳妥处理豇豆事件，努力克服强降雨灾害的影响，全年实现农业总产值46.6亿元，同比增长6.1%。积极壮大海洋产业，基本建成了南山港区一期工程，在继续发展外海远洋捕捞的同时，推动近海海洋牧场规划建设，推广优质品种养殖，加快了水产品加工业和休闲渔业发展。

三、积极推进节能减排和生态环保工作，不断增强可持续发展能力。荔枝沟落笔洞区污水处理厂建设接近尾声，海棠湾污水一厂、二厂、创新创意产业基地南区污水处理厂一期、市区污水管网改造、城市中水回用工程以及亚龙湾冰蓄冷区域供冷站示范项目总体进展顺利。积极推广使用太阳能产品，在全省率先拆除了全部粘土实心砖生产线，推广应用新型墙体材料，鼓励使用建筑节能技术，节能减排工作取得新成效。《三亚生态市建设规划》和《三亚市环境保护规划》已通过评审并开始实施，强化了海洋环境监察、保护，完成了沿海基干林带恢复建设工程，积极推进三亚湾、鹿回头湾等海湾生态环境修复和保护，推进受损山体绿化恢复、退耕还林等工程，生态保护和环境治理进一步加强。

四、积极推进城市规划建设，努力提升城市管理水平。根据国际旅游岛建设的要求，对新版城市总规进行了全面修编，已获省政府批准。完成了凤凰机场二期扩建工程，东环铁路三亚站、亚龙湾站已启用，三亚绕城高速公路路基工程以及亚龙湾连接线、天涯连接线已建成，完成了火车站出口路和新风路加宽改造等工程，打通了团结路，完成了部分道路、交叉路口、交通转盘的改造。西部供水工程、民用天然气输配管网、城乡电网建设工程进展顺利，三亚湾绿地改造已完成。启动了数字城市综合监管项目和综合管理系统工程建设。将部分城市管理权限委托给河东、河西、凤凰等区镇，强化了属地管理作用。新建、改建了6个停车场，完成了三亚湾路和解放路62个公交候车亭港湾交通改造，调整了6条公交线路，更新了一批公交车辆，大力开展道路交通环境综合整治，改善了城市交通状况。开展了打击违法违章建筑的“铁锤行动”，遏制了违法抢建之风。新购置了一批环保车辆，完成了4座垃圾中转站建设并投入使用，增设了公共厕所、果皮箱等环卫设施，大力开展卫生环境综合整治，巩固了创卫成果。

五、积极推进农村建设发展，加快农村改革步伐。大隆水库灌渠工程、三亚西河槟榔河段治理工程进展顺利，福万、抱古等16宗病险水库除险加固、8宗病险水库应急加固以及三亚东河防潮(洪)堤等工程已完工，水毁工程修复、渠道建设和清淤维护、田洋整治等农田水利建设扎实推进。投入1亿元新建129个文明生态村，农村道路建设、饮水安全、扶贫攻坚等工作取得新成效。农村土地制度改革试点工作稳步推进，集体林权制度改革主体任务已全面完成，通过林改放活了林木经营权，落实了处置权，规范了集体山林承包、流转，建立了林业贷款财政贴息、林权抵押贷款制度。

六、积极推进体制机制创新，加大对外开放力度。2010年，我市已被确定为全国37个“国家服务业综合改革试点区域”之一。大力推进基本公共卫生服务项目和重大公共卫生服务项目，推进公立医院改革试点，从去年1月1日起在全市基层医疗机构全面实施基本药物制度。积极探索推进强镇（区）扩权财政体制改革，全市社保费征管及地税农税体制调整改革已完成。继续实施财政惠民补贴资金“一卡通”改革，全年发放惠民补贴资金990万元。崖城镇众树农村资金互助社已开业，新型农村金融机构准入工作取得实质性进展。充分利用“珠洽会”、上海世博会海南周活动、海南（香港）现代服务业推介会等平台，积极组织招商活动，2010年我市实际利用外资1.98亿美元，同比增长13.3%。

七、积极推进民生事业发展，不断改善人民生活。2010年，“同心家园”三到六期等7个保障性住房项目已分期分批开工，共建设住房9966套，全市完成保障性住房审核分配四批次4238户，发放住房租赁补贴822万元。全市新增城镇就业岗位36850个，农村富余劳动力实现转移就业18781人，城镇登记失业率为1.55%。认真实施事业单位绩效工资改革，进一步提高了教师等事业单位人员工资水平和离退休人员生活补贴。积极推进新型农村社会养老保险试点、城镇老年居民基本养老保险等工作，扩大了社保覆盖面，提高了城乡低保标准，在全省率先启用价格调节基金对全体城乡居民发放临时物价补贴。积极推进新一轮中小学布局调整和规划建设，中小学校舍安全等工程进展顺利，中等职业教育、高等教育发展进一步加快。市中医院二期工程等医疗卫生项目顺利推进，在市级5家医院和11个农村卫生院之间建立了对口帮扶关系。有效应对强降雨灾害的影响，努力将损失降到最低限度，灾后迅速拨付特大暴雨防汛救灾资金8377万元，救灾安置和恢复重建工作顺利推进。严厉打击毒品犯罪、“两抢一盗”犯罪，大力开展扫黄禁赌专项行动，切实加强公共场所、特种行业和流动人口管理，建立起各警种联合社会面防控体系，社会治安环境明显改善。食品药品安全和产品质量保障工作进一步强化，安全生产形势平稳趋好。积极推行信访代理制度，努力将矛盾化解在基层，有效预防和妥善处置群体性事件，社会保持和谐稳定。

八、积极推进政府自身建设，着力优化政务环境。完成了新一轮政府机构改革。市政府政务服务中心正式运行，目前全市共有729项行政审批（服务）事项进驻，至2010年底共受理行政审批（服务）事项15191件，按时办结率达到98%，提前办结率达到90.8%。认真执行市人大及其常委会的决议、决定，自觉接受市人大的法律监督、工作监督和市政协的民主监督。认真办复市人大代表建议、批评和意见76件、市政协委员提案102件，办复率达到100%，满意和基本满意率达到93%，切实解决了一批人民群众关注的热点问题。认真开展反腐倡廉“制度执行巩固年”活动，进一步完善并严格落实重要岗位和重点领域权力运行制度，对海棠湾、三亚绕城高速公路等重大项目实行了全程监督。加大行政问责力度，坚决查处违纪违法案件，政府廉政建设进一步加强。

与此同时，我市积极开展国防教育、国防动员、民兵预备役工作，扎实推进双拥共建工作，军政军民团结进一步巩固。民族宗教、外事侨务、人口计生、新闻出版、统计、档案、史志、口岸、地震、人防等工作取得新成绩，工会等工作进一步加强，红十字、妇女儿童、老龄和残疾人等事业也取得了新的进步。

各位代表，过去一年的发展成绩来之不易，这是省委、省政府和市委正确领导的结果，是全市人民共同奋斗的结果，是市人大、市政协和社会各界监督支持的结果。在此，我谨代表市人民政府，向全市各行各业的建设者，向人大代表、政协委员，向各民主党派、工商联、无党派人士以及各人民团体，向驻三亚解放军、武警官兵、公安司法干警，向中央和省驻三亚各单位，向所有参与、支持、关心三亚建设发展的海内外朋友表示衷心的感谢和崇高的敬意！

回顾过去的一年，我们也清醒地看到，三亚发展的过程中仍然面临着不少困难和挑战，政府工作还存在一些问题和不足，主要表现在以下方面：经济总量仍然较小，经济发展中房地产业所占比例偏大，产业结构抗风险能力较弱；旅游要素国际化改造有待加快，旅游产品的国际竞争力急需增强；违法建设行为严重影响城市发展，整治任务十分艰巨；旧城改造进展缓慢，城市功能和管理水平与国际旅游岛建设的要求差距较大，尤其是城市交通、城市排水、环境卫生、社会治安、旅游市场秩序等方面的问题较多；教育、医疗等社会事业发展滞后，相对于经济和财政收入增长速度，城乡居民收入增长仍然较为缓慢，且城乡差距仍在继续拉大；政府工作人员整体素质和能力有待增强，政府执行力和工作效率有待进一步提升。对于这些问题，我们将积极采取有效措施加以改进和解决，努力把政府各项工作做得更好。

“十一五”发展回顾和“十二五”目标任务

2010年各项发展目标的实现，为我市“十一五”时期划上了圆满的句号。五年来，我市深入贯彻落实科学发展观，积极应对全球金融危机等重大挑战，努力打造实力三亚、活力三亚、美丽三亚、文化三亚、和谐三亚，全面超额完成了“十一五”规划的主要目标和任务。全市生产总值年均增长18.2%，继2007年突破100亿元之后，2010年又突破200亿元。去年，全市固定资产投资、社会消费品零售总额、地方财政一般预算收入、城镇居民人均可支配收入、农民人均纯收入已分别达到2005年的6.2倍、2.9倍、5.8倍、2.1和1.9倍。积极统筹城乡发展，城市建成区面积从2005年的25平方公里扩大到45平方公里，城镇化水平从47.3%提高到56.5%，创建文明生态村701个，一批重大基础设施项目相继建成或开工，提前一年完成了“十一五”节能减排任务。政府改革和自身建设、市场经济体制改革、财政投融资体制改革、农村综合配套改革取得新成效，对外开放进一步扩大，发展活力不断增强。制定实施了《三亚市2008―2013年重点民生项目发展规划》，“十一五”时期累计投入重点民生项目资金76.2亿元，就业工作进一步加强，社保覆盖面不断扩大，住房保障体系逐步建立健全，科教文卫体等社会事业全面发展，人民生活水平显著提升。

各位代表，我们欣喜地看到，“十一五”时期已成为三亚经济增长最快、发展质量最好、改革力度最大、人民群众得到实惠最多的时期。这些成绩为三亚“十二五”时期的发展打下了坚实的基础。根据《中共三亚市委关于制定国民经济和社会发展第十二个五年规划的建议》，市政府编制了《三亚市国民经济和社会发展第十二个五年规划纲要(草案)》（以下简称《纲要（草案）》），现已提请大会审议。《纲要（草案）》简要分析了“十二五”时期我市所面临的发展形势，提出了今后五年全市经济社会发展的指导思想、基本原则、奋斗目标和主要指标，突出了科学发展的主题和加快转变经济发展方式的主线，并对“十二五”时期的战略重点和主要任务进行了全面规划，这里分六个方面作简要说明：

一、加快产业发展，做强经济实力。一方面，着力丰富旅游产品体系，优化旅游发展空间格局，加快旅游要素国际化改造，加强旅游管理和服务，提升旅游核心竞争力，努力缩小与世界水平的差距。另一方面，继续推进现代农业、房地产业、商贸、商务和金融业、创新创意产业、海洋产业以及文化体育产业发展，打造特色产业体系，增强经济发展的稳定性。

二、优化发展格局，完善基础条件。全面实施新版城市总规，构建功能定位明晰、发展导向明确、城镇体系完善的发展格局。坚持升级改造与增量新建并重，适度超前，积极实施综合交通运输体系建设、市政基础设施建设、水利设施建设等重点工程，提升城市信息化水平，完善基础条件，提高承载能力。同时，按照城乡统筹、全面小康的要求，进一步推进新农村建设，继续实施村庄整治，不断完善农村基础设施和公共服务。

三、强化城市管理，提升城市品位。创新城市管理和社会管理体制，加强社区建设，发挥居（村）委会的基础管理作用。建立健全数字化城市管理系统，继续完善城市管理市场化机制。引导基层自治组织、社会团体、行业组织和其它非政府组织利用各自优势，提供公共服务，参与社会管理。进一步完善社会治安防控体系，严格落实安全生产责任制，加强应急管理机制建设，提高社会治安和公共安全水平。

四、建设生态文明，实现绿色发展。积极构建低碳发展模式，打造全国生态文明建设示范区。大力实施低碳旅游示范、绿色能源推广、绿色照明推广等工程，发展低碳产业，建设低碳城市。统筹安排城乡建设用地和生活、生产、生态用水，提高土地集约化利用水平和水资源综合利用效率。综合运用各种经济杠杆，有效引导社会、企业节约资源。强化三大生态功能区建设，实施海洋生态综合管理，加大生态恢复和保护力度。进一步完善城乡污水处理设施，加强污染防治、环境保护和综合治理。

五、深化体制改革，扩大对外开放。依托国际旅游岛和“国家服务业综合改革试点区域”建设，开展多领域体制机制改革。着力推进旅游管理体制和运行机制改革，继续深化国企改革、农村综合配套改革、行政管理体制改革，强化财政公共服务职能，加快社会事业改革，促进农垦与地方融合。积极开展国际交流与合作，引导外资投向高新技术产业和现代服务业，进一步加强与泛珠三角等区域和三亚周边市县的合作。

六、全力改善民生，构建和谐社会。坚持以人为本，把改善民生作为政府工作的着力点和落脚点，努力建立覆盖城乡、可持续的基本公共服务体系，千方百计促进就业，着力提高社保水平，加快教育、卫生事业发展，加强保障性住房建设，努力使全市人民学有所教、劳有所得、病有所医、老有所养、住有所居，建设三亚人民的幸福家园。

全力做好2011年政府工作

2011年是实施“十二五”规划的第一年，也是国际旅游岛建设关键之年，做好今年各项工作，对实现“十二五”规划良好开局具有十分重要的意义。根据省委、省政府的总体要求和市委的部署，2011年市政府工作的总体思路是：认真贯彻党中央、国务院和省委、省政府的要求，全面落实市委五届七次全会精神，以邓小平理论和“三个代表”重要思想为指导，以科学发展为主题，以加快转变经济发展方式为主线，紧紧围绕国际旅游岛建设，努力提升旅游产业发展质量，加快产业结构优化，加强节能减排和生态环保工作，着力抓好城市建设管理，进一步推进新农村建设，不断深化改革开放，千方百计改善民生，促进全市经济社会又好又快发展，确保“十二五”规划开好局、起好步。

根据上述思路，综合分析发展环境和自身条件，2011年我市经济社会发展的主要预期目标是：全市生产总值增长13%，全社会固定资产投资增长20%（按统计新口径计），社会消费品零售总额增长18%，地方财政一般预算收入增长14%，旅游接待过夜游客增长12%，城镇居民人均可支配收入增长10%，农民人均纯收入增长12%，居民消费价格总水平涨幅控制在4%左右，城镇登记失业率控制在3%以内，单位生产总值能耗、主要污染物排放量削减完成省下达的任务。为实现上述目标，今年要重点抓好以下几个方面的工作：

一、加快旅游要素国际化改造，在国际旅游城建设上取得新突破

国际旅游岛建设已经进入第二年，有关工作将进入抓落实、见成效的阶段。今年我们要努力加快旅游要素国际化改造，提升旅游产业发展质量。

（一）积极推进海棠湾等片区开发。进一步加快海棠湾市政工程和滨海带酒店建设，确保今年有6家酒店开业，大力推进解放军总医院海南分院、梦幻娱乐不夜城、惠普云计算动漫产业、国家海岸湿地公园、国际风情小镇、国际会展中心、国际艺术中心等一批重点项目建设。加快亚龙湾二期开发、红塘湾旅游度假区以及凤凰岛开发、鹿回头半岛开发等项目进度，着力完善各片区配套设施和功能。

（二）进一步优化旅游产品结构。积极开展邮轮母港前期工作，争取更多邮轮停靠，加快推进三亚河游艇基地等项目，完成《三亚市游艇产业发展战略规划和布局控制性详规》的编制，成立三亚游艇游船（帆船）协会，大力开发海洋旅游系列产品。积极推进亚龙湾热带森林公园旅游区、三亚槟榔河文化旅游区、泛南山国际旅游区以及落笔洞国际康体养生中心等项目建设，开工建设南新温泉康体疗养中心一期项目，积极引进高水平的康复、疗养机构，促进森林旅游、文化旅游、温泉养生、康复疗养旅游发展，继续围绕革命老区发展红色旅游，丰富三亚旅游产品体系。

（三）着力提升旅游服务和管理水平。积极推进三亚市游客到访服务中心建设，启动第二批游客到访服务站（亭）建设，进一步完善旅游公共服务体系。加快“天涯通”旅游信息服务体系建设，推动建立旅游诚信商家联盟，继续实施品质服务计划。积极发挥旅游行业协会的作用，分阶段将部分旅游服务标准化工作移交给协会。继续推进大型旅行社网点建设，支持旅游企业做大做强。积极引进旅游管理人才，加强旅游从业人员培训。继续开展旅游市场环境整治，严厉打击旅游“四黑”，开展“零负团费”专项治理，认真查处各类违法违规经营行为，加快探索建立规范的旅游长效管理机制。

（四）加强旅游市场宣传促销。充分利用国际旅游岛品牌，利用三亚国际旅游形象标识、沃尔沃帆船赛等载体，积极创新旅游营销方式，着力提升营销质量。在重点国际客源国设立联络处并委托建立旅游外文网站，在国内重点客源城市设立营销专柜，开展阵地式宣传促销。有效稳固俄日韩市场，拓展欧洲市场，全面启动美国、加拿大市场营销，继续深化港澳台市场营销与合作，积极开拓中东等新兴市场。

二、大力推进产业结构优化，在转变经济发展方式上取得新突破

产业结构优化是转变经济发展方式的重要内容。我们要在提升旅游和房地产业发展质量的同时，加快培育新的产业经济增长点。

（一）保持房地产业健康可持续发展。认真贯彻落实国家调控政策，进一步完善和切实落实三亚楼市调控实施细则，加强土地资源管理，严格控制规划指标，有效推动房地产业“高端、适度、有序”发展。鼓励开发高品质的星级宾馆、度假村等项目，稳步发展度假居住型房地产。着力规范产权式度假酒店等项目开发管理，规范中介机构发展和家庭旅馆经营，完善房地产项目的停车场所、商业服务、文体活动、教育医疗、社区服务等配套设施，营造宜居环境，创造就业岗位，拉动消费需求。强化房地产市场监管，落实差别化信贷政策，抑制投资投机性需求。

（二）加快培育创新创意产业。大力推进创意产业园征地拆迁工作，加快园区基础设施和安置区建设，完成标准厂房建设，开工建设科技孵化及创业服务中心综合楼。认真落实产业发展和项目建设优惠政策，实施项目跟踪服务，加快中兴通讯三亚研发中心等项目进度，积极开展联想产业园等新签约项目前期工作，力争有部分项目早见成效。加快市高新技术产业园基础设施建设，保障海南动漫产业基地等项目顺利推进。

（三）着力推进商贸产业发展。继续开展境外旅客购物离境退税政策试点工作，加快海南国际免税城、亚龙湾奥特莱斯商业综合体等项目进度，积极推进亚龙湾、三亚湾、槟榔河、吉阳（田独）旅游商业街改建和新建工程，完善旅游商贸体系。同时，加快实施《2010―2015年商贸项目建设计划》，健全城市商业网络体系，继续推进农贸市场升级改造，推进“万村千乡市场”工程，鼓励、支持大型百货商店和超级市场在居住小区和各镇设立连锁店，进一步扩大城乡居民消费。

（四）积极实施“文化强市”战略。加大文化体制改革力度，继续开发和传承黎、苗少数民族文化，努力打造本地特色文化产品，继续引进有实力的文化产业项目。完成三亚市体育中心建设并投入使用，积极推进奥林匹克湾、南中国海影视文化生态公园、沃尔沃帆船赛经停港基地、崖城历史文化名镇保护与整治等项目，加快策划推进市科技馆、博物馆、音乐厅、艺术馆、会展中心和中国衣食父母园等项目。大力开展国际文化交流活动，举办好世界沙滩排球巡回赛等文体赛事，积极引进和培育品牌会展。继续推进城市社区和农村地区文体设施建设，积极开展群众性文化活动和全民健身活动。

（五）大力发展热带特色现代农业。稳定粮食生产，提升冬季瓜果菜生产和管理水平，大力扶持热带水果、热带兰花、南繁育种等特色产业发展，抓好畜禽养殖小区示范点和种苗繁育体系建设。按照“设施农业+产业化”的思路，加快设施农业和农产品基地建设。继续推广“企业+合作社+基地+农户”的经营管理模式，提升农产品附加值，加强特色品牌建设。建立健全市、镇、村一体化农技服务网络，大力推广测土配方施肥和病虫害绿色防控技术，加强农业投入品监管，完善农产品质量检测体系。积极扶持崖城、海棠湾农产品批发市场建设，扶持冷藏保鲜企业发展，打造农业产、供、销整体产业链。实施“农超对接”建设项目，支持大型连锁企业与农民专业合作社建立长期稳定的产销合作关系。

（六）推动产业发展向海洋迈进。继续推行海域使用权公开招标、拍卖制度，推进东锣、西鼓无居民海岛项目开发。启动崖州三港经济区项目，加快建设崖州中心渔港。继续实施渔业综合补给船项目，支持渔船更新改造，启动南沙渔场母船作业群项目，发展外海远洋捕捞。实施《三亚市近海海洋牧场建设规划》，加强以人工鱼礁与增殖放流为主的海洋牧场建设。引进推广优质水产品种，重点发展深水抗风浪网箱养殖和贝类底播养殖，继续培育水产品加工龙头企业。推进休闲渔业示范点建设，适应休闲渔业和游钓业市场需求。

三、坚持可持续发展，在生态文明建设上取得新突破

加强节能减排和生态环保工作，是转变经济发展方式的重要内容。我们要在加快发展的同时继续保护好三亚的生态环境，维护好三亚的比较优势。

（一）加强节能减排工作。切实落实节能减排政策，认真实施差别电价等措施，制订出台三亚市节能管理暂行规定。加快完善基础设施，推进海棠湾污水一厂、二厂、创新创意产业基地配套污水处理工程、市区污水管网改造和各镇污水管网配套等工程建设。积极开展可再生能源建筑应用城市示范工作，大力推广太阳能、沼气利用，完成1个兆瓦级太阳能屋顶并网发电示范项目建设，力争完成150万平方米的太阳能热水系统建筑应用任务。新建2―3个新型墙体材料厂，加快推进亚龙湾冰蓄冷区域供冷站示范项目的应用。积极发展循环经济，加快城市中水回用工程建设，建立健全再生资源回收体系。

（二）加强生态环保工作。认真组织实施《三亚生态市建设规划》、《三亚市环境保护规划》、《三亚市海洋环境保护规划》和《三亚市林地保护利用规划》，大力推进生态市建设。加快实施三亚河生态整治、月川湿地生态保护治理等工程，加强饮用水水源保护区规划建设，抓好受损山体绿化恢复、退耕还林、三边林建设、护林防火以及森林生态补偿试点等工作。继续开展“蓝丝带”海洋环境保护，做好三亚国家级珊瑚礁自然保护区调整工作，加强重点海湾海洋环境和海岸工程监管，推进鹿回头湾生态环境修复和岸滩整治，实施三亚湾滨海绿带工程。积极开展环保专项行动，强化汽车尾气等污染治理。

四、加快城乡建设步伐，在筑牢发展基础上取得新突破

与国际旅游岛建设的要求相比，我市的城市功能和管理水平还有很大差距。我们要大力推进基础设施建设，完善城市功能，加强城市管理。同时，继续开展城乡一体化建设试点工作，提升农村地区建设发展水平。

（一）大力推进基础设施建设。启动《三亚市市政基础设施专项规划》编制工作，积极落实三亚市综合交通设施建设五年实施计划，在城市外围，加快推进凤凰机场三期扩建前期工作，力争三亚绕城高速公路在10月底前建成通车，积极推进海榆东线榆林至吉阳（田独）段道路改造、凤凰镇至西线高速公路道路、崖城镇过境公路及旧桥连接线等工程建设；在城市市区，完成三亚湾新城东路、凤凰水城规划中心路、新风桥重建等工程，加快推进全市社区道路改造工程(一期)，开工建设解放四路延伸段、春光路、月川和儋州社区市政道路以及三亚大桥重建、胜利路改造等工程，启动和加快三亚湾路至鹿回头跨海大桥、市公共汽车东站、西站以及轨道交通等项目的前期工作。同时，要在年底前完成《三亚市中心城区与滨海地区停车专项规划》并付诸实施。开工建设凤凰路等道路排水项目，继续推进西部供水工程、城乡电网建设工程，进一步完善天然气输配管网，推进城市公厕体系、南边海垃圾中转站、村镇垃圾中转站及垃圾收集转运系统建设，开工建设城市灯光夜景三期等景观工程。

（二）加快实施旧城改造。在积极推进城市新区建设的同时，着力加快旧城改造工作进度。抓紧实施阳光海岸、月川等片区控制性详细规划，加快推进水居巷、下洋田、天阔广场、榕根以及吴春园等旧城改造，促进阳光海岸、月川、南边海等区域的改造项目有序实施。继续结合旧城改造工作完善城市交通、水电、燃气、通讯、商业、教育、医疗和社区服务等配套设施，加快打造“半小时便利生活圈”。统一新区建设和旧城改造标准，严把规划设计和施工许可关，配套服务设施与住宅建设项目必须同步规划、同步设计、同步审批、同步建设、同步验收、同步交付使用，杜绝挤占、变更配套服务设施的行为。逐步发展城市地下设施，提高地下空间的利用率。

（三）努力提升城市管理水平。进一步探索推进城市管理权限下放，大力加强社区建设，加快实施社区居委会办公与公共服务用房项目。积极开展数字城管工作，争取在10月前正式启用数字城市综合管理系统。大力强化城管执法队伍建设，加强执法人员培训，提高执法水平，坚持依法执法、文明执法。进一步规范和优化灯控系统、道路交通标志标线、交通护栏等设施设置，加快推进城乡公交一体化建设。继续开展道路交通环境综合整治，维护交通秩序。坚决打击违法建设行为，力争在7月前将应当拆除的违法违章建筑全部拆除。进一步推进城乡宅基地和房屋确权登记发证工作，打击非法买卖集体土地的行为，不断完善建房报建制度，引导居民依法报建。建立健全预警机制，由区镇属地承担第一责任，全面加强对新增违法违章建筑的巡查监管，做到及时发现、及时处置，将问题解决在萌芽阶段。认真实施三亚市国家卫生城市长效管理机制和细则，加强社区和单位、建筑工地以及城中村和城乡结合部管理，不断优化城市卫生环境。

（四）进一步推进新农村建设。加快实施大隆水库灌渠工程、赤田水库下游河道防护工程、宁远河综合整治工程以及小型病险水库除险加固工程，着力推进农田水利建设。进一步巩固现有文明生态村建设成果，积极推进新增文明生态村建设，修建40公里新农村道路，完善多功能服务中心、文化室、篮球场、休闲广场等公共文化设施，抓好饮水安全、改水改厕、扶贫攻坚等工作，不断改善农村生产生活环境。进一步深化农村综合配套改革，加强农村土地流转指导、协调、服务和管理。着力抓好林权配套改革，进一步规范林地、林木流转，建立支持集体林业发展的公共财政制度，推进林业投融资改革，加强林业社会化服务体系建设。继续实施财政惠民补贴资金“一卡通”，认真落实各项惠农政策。促进新型农村金融机构准入工作稳步开展，支持农村信用社改革，不断完善小额信贷、农户联保贷款和农业保险等金融服务。

五、不断深化改革开放，在增强发展活力上取得新突破

充分利用国际旅游岛建设等方面的优惠政策，着力推进重点领域和关键环节改革，加快体制机制创新，提高对外开放水平，增强发展活力和动力。

（一）积极推进体制机制改革。加快制定和大力实施服务业综合改革试点工作方案。严格执行产权交易制度，规范国有资产流转、国有产权转让、国有资产评估和不良资产核销等工作，抓好改制、关闭退出企业的资产处置和职工安置。整合优质资源，建立健全良性的投融资运作机制，在优势产业方面着力培育龙头企业和上市主体。加强企业工资分配管理，严格执行国企负责人薪酬管理相关规定。加快推进财政科学化、精细化管理，推进市本级财政预算项目绩效评价体系建设。优化财政支出结构，进一步向民生事业、公共服务、基础设施、农村建设等方面倾斜。充分发挥财税政策的导向作用，引导产业发展和社会投资，调节社会成员收入分配。进一步完善国有资源有偿使用机制，加强海域资源、矿产河砂等公共资源的有偿使用收入管理。

（二）提高对外开放水平。继续实施“走出去、引进来”的开放战略，积极组织参加第七届“珠洽会”、第十五届中国国际投资贸易洽谈会、第十三届中国国际高新技术成果交易会等活动。以产业规划为指导，不断优化外商投资结构。继续实施科技兴贸战略，积极发展水产品、热带花卉、蔬果等热带优势农副产品，着力培育东吉、远跳等龙头企业，引导企业向深加工领域发展，提高出口产品附加值。努力创造有利条件，推动服务贸易与货物贸易共同发展。加强口岸基础设施建设，提升口岸服务水平，营造良好的外贸发展环境。

六、扎实推进各项民生工程，在提升人民生活水平上取得新突破

加快实施重点民生项目发展规划，努力完善基本公共服务，扎扎实实为人民群众办好事，办实事，让城乡居民得到更多实惠。

（一）加快保障性住房建设分配。在大力推进“同心家园”三到六期等项目建设的同时，今年要新开工建设经济适用房2400套、公共租赁房1000套、廉租住房800套，实施城市棚户区改造300套、农村危房改造1590套。抓好保障性住房审核分配，完成1772户廉租住房、5594户经济适用房的审核并分配到户。加快研究制定公共租赁房的申报审核方案，启动公共租赁房申报工作。继续做好廉租补贴发放工作，切实提高保障对象的支付能力。

（二）着力加强就业工作。继续扶持中小企业发展，扶持服务业态发展，积极开发就业岗位。大力推进公共就业三级服务平台和服务信息化建设，开展公共就业服务专项活动，实施职业培训计划，重点围绕高校毕业生、下岗失业人员、零就业家庭和失地农民开展就业服务和援助工作。加快创业孵化实训基地配套建设，积极探索开展小额担保贷款业务，鼓励和扶持自主创业。继续实施“阳光工程”，力争实现农村富余劳动力转移就业13200人。

（三）不断完善社会保障体系。继续做好社保扩面征缴工作，进一步提高新型农村社会养老保险参保率，逐步在全体城镇居民中开展养老保险试点工作。进一步加快基本医疗保险制度建设，将破产、改制国企退休人员纳入城镇从业人员基本医疗保险保障范围。以解决大病费用负担为重点，逐步提高医保待遇水平，扩大门诊特殊病种范围，提高报销比例。继续实施城乡医疗救助，积极探索困难群众“门诊（药店）医疗卡”制度。进一步提高城乡低保和农村五保保障标准。继续加强价格监测预报预警和价格信息发布工作，建立物价上涨财政联动机制，根据价格波动情况，科学合理地调控价格，实施价格救助。

（四）大力发展教育事业。完成2个区镇中心幼儿园的建设，对私立幼儿园进行整顿规范。继续推进中小学布局调整，加强义务教育阶段学校标准化建设，实施中小学校舍安全工程，着力改善农村薄弱学校的办学条件。强化校长和教师队伍培训，进一步推动义务教育教师资源均衡配置。积极扩大高中阶段教育规模，强化中等职业技术学校招生自主权，促进中等职业教育和普通高中教育协调发展。继续完善义务教育经费保障机制，切实落实“两免一补”、“三免一补”等政策，做好生源地信用助学贷款工作，建立健全高中困难学生资助体系。积极推进琼州学院三亚校区、三亚航空旅游职业学院三期等项目建设，大力发展特色学科和专业，进一步促进高等教育为经济社会发展服务。

（五）提高医疗卫生服务水平。加快推进市中医院二期工程，开工实施市人民医院改扩建二期、市中医院三期工程和三亚市妇幼保健院整体搬迁（新址）建设项目。进一步完善基层医疗卫生服务体系，加强社区卫生服务中心、服务站和农村卫生院建设，抓好基层设备配备、人才建设。围绕医院管理体制、运行制度、人事制度、分配制度、经费保障制度，加快公立医院改革步伐。积极推进基层医疗卫生体制综合改革，继续实施基本药物制度，在市级5家医院和11个农村卫生院之间认真开展对口帮扶。扎实推进基本公共卫生服务项目和重大公共卫生服务项目。鼓励和引导社会资本举办医疗机构，扩大医疗服务供给，提高服务质量，完善服务体系。

（六）维护社会和谐稳定。大力加强精神文明建设，深入开展法制宣传教育，提高市民文明水平和法律素质。进一步加强矛盾纠纷排查化解和信访积案化解工作，健全信访信息报送制度，强化信访事项督查督办，力争将矛盾化解在萌芽状态，有效预防和妥善处置群体性事件。全面落实安全生产责任制，切实加强事故多发行业和领域专项整治，严防重大事故发生，降低一般事故发生率。继续强化食品、药品安全监管工作，完善城镇餐饮服务监管体系。加强社会治安综合治理，继续实施“110”警务岗亭上街工程，完善社会治安防控体系，开展社会治安清查整治，提高流动人口管理水平。继续保持对毒品犯罪的严打高压态势，持续开展打击“两抢一盗”等多发性侵财犯罪专项斗争。加快制定重大公共安全事件和突发事件应急联动预案，建立健全数字化应急指挥平台，按照“专兼结合”的原则加强应急队伍建设，积极开展人员培训和应急演练，增强针对突发事件的应急救援能力。进一步加强国防动员和民兵预备役工作，大力支持国防建设，广泛开展双拥共建活动。加快发展妇女儿童、老龄、残疾人以及红十字、福利事业，继续做好民族宗教、外事侨务、人口计生、新闻出版、防震减灾、气象、统计、档案、史志等工作。

七、努力加强政府自身建设，在优化发展环境上取得新突破

积极适应发展形势的需求，进一步转变政府职能，注重制度建设和管理创新，不断增强服务能力，努力建设高效政府、法治政府和廉洁政府。

（一）大力推进高效政府建设。继续深化行政审批制度改革，依托市政府政务服务中心，对行政许可和行政审批事项进一步进行梳理，简化办事程序，压缩办理时间，完全达到“三集中”的要求。筹划设立重大项目委员会，建立健全并联审批制度，实行联审联办考评制度。在海棠湾实践的基础上，加大力度推进强镇（区）扩权改革，强化区镇在社会管理、公共服务等方面的权限和责任。按照机构、人员、财力与事权相匹配的原则，抓好区镇机构设置、人员配备和财政体制改革。逐步推行文职雇员制，优化政府机关人员结构。不断完善政府层级管理，强化工作督查尤其是重大决策和重点工作跟踪督办，认真开展绩效考评，切实落实各项问责制度。

（二）大力推进法治政府建设。自觉接受人大法律监督、工作监督和政协民主监督，坚决执行市人大及其常委会的决议、决定，认真听取各民主党派、工商联、各人民团体和各界人士的意见、建议。坚持依法行政，规范政府行政行为，积极推进科学决策、民主决策，努力健全重大决策听证、重要事项公示等制度，注重公众参与，保障人民群众充分表达意见的权利。进一步完善行政执法体制，规范行政执法程序，依法细化、量化自由裁量权。加强行政复议工作，依法纠正不当和违法行政行为。不断完善和切实落实政务公开制度，大力推进财政资金公开、审计结果公开，尤其是涉及民生的重点资金、重点项目公开，着力扩大民生与公共服务类信息公开。

（三）大力推进廉洁政府建设。进一步加强廉政教育和廉政制度建设，严格执行廉洁从政各项规定，深化源头治腐工作，不断完善惩防并举、注重预防的廉政体系。强化对各项重大决策、重点工作的行政监察，认真治理征地拆迁、项目审批、税收征管、房地产开发、工程招投标等领域的突出问题。尽快启动市政府政务服务中心公共资源交易大厅运行，将全市各类公共资源交易活动纳入统一管理，实现“管办分离、集约交易、职责分开、集中监管”。严厉查办官商勾结、权钱交易的案件。着力解决发生在人民群众身边的腐败问题，维护好人民群众最直接、最现实的利益。

各位代表，2011年是“十二五”规划开局之年，三亚面临着新的机遇和挑战。让我们紧密团结在以胡锦涛同志为总书记的党中央周围，深入贯彻落实科学发展观，在省委、省政府和市委的坚强领导下，团结一致，开拓创新，真抓实干，进一步推动三亚又好又快发展，努力实现“十二五”规划良好开局，以优异的成绩迎接中国共产党成立90周年！
